# Lipid Extraction and Cell Disruption Methods for Improving Biodiesel Production by *Scenedesmus sp*.

**DOI:** 10.3390/microorganisms14040731

**Published:** 2026-03-24

**Authors:** Mᵃ Pilar Patón Raya, Mᵃ Lourdes Martínez Cartas, Sebastián Sánchez

**Affiliations:** 1 Department of Chemical, Environmental and Materials Engineering, Science & Technology Campus (Linares), University of Jaén, Avda. de la Universidad s/n, 23700 Linares, Spain; mppaton@ujaen.es (M.P.P.R.); ssanchez@ujaen.es (S.S.); 2 University Institute of Research on Olive and Olive Oils (INUO), GEOLIT Science and Technology Park, University of Jaén, 23620 Mengíbar, Spain

**Keywords:** microalgae, *Scenedesmus sp.*, cellular disruption, microwaves, ultrasounds, lyophilisation, autoclaving, electroporation, biodiesel

## Abstract

Lipid recovery efficiency from microalgal biomass is a critical factor in the commercial viability of biodiesel. *Scenedesmus sp*. presents a robust cell wall that necessitates the evaluation of specialised disruption techniques to enhance intracellular lipid release and subsequent fuel quality. This study investigated the efficacy of five cell disruption methods—microwaves, ultrasonication, lyophilisation, autoclaving, and electroporation—integrated with three distinct extraction procedures: cold extraction, Soxhlet extraction system, and microwave-assisted extraction. The qualitative and quantitative impacts of these treatments were assessed by analysing the fatty acid methyl ester (FAME) profiles via gas chromatography (GC) following transesterification. The highest total lipid yield (88.97%) was achieved through a combination of microwave disruption and Soxhlet extraction. However, the maximal proportion of methyl esters was obtained when ultrasonication was paired with microwave-assisted extraction (97.64%). Surface analysis using scanning electron microscopy (SEM) of samples subjected to different disruption procedures could support the conclusions. Similarly, when the microalgal biomass was lyophilised beforehand, microwave extraction increased the oleic acid content. These results indicate that the choice of disruption and extraction protocols significantly influences both lipid recovery rate and the proportion of fatty acids in the chemical composition of microalgae. Tailoring these processes is essential for optimising the fatty acid profile for high-quality biodiesel production.

## 1. Introduction

In the near future, renewable liquid biofuels will replace petroleum-derived transport fuels. Microalgae are a promising alternative, with oil content that may exceed 80% (*w*/*w*), compared with 5% for the best agricultural oil crops [1]. Microalgal biotechnology has high potential for biodiesel production because of the significant increase in microalgae lipid content [2]. Ensuring access to affordable, reliable, sustainable, and modern energy for all requires rapidly increasing the use of renewable energy in electricity, heat, and transport. Target 7.2 of the Sustainable Development Goals (SDGs) is to “increase substantially the share of renewable energy in the global energy mix” by 2030 [3], contributing to strategies to achieve a climate-neutral economy by 2050 [4]. To achieve these goals, it will be necessary to develop more and better biofuel production methods that allow for cost-effective generation. In order to reach these objectives, it will be necessary to have more and better procedures for obtaining biofuels that allow them to be generated in a profitable way. Biodiesel generation through the transesterification of triglycerides from the fatty matter extracted from microalgal biomass is a promising alternative [5,6,7].

The lipids required for the transesterification process involve a prior stage of biomass separation with extraction processes [8]. Biodiesel can be obtained from biomass using a one-step direct transesterification method [9] or sequentially in two stages of extraction-transesterification [10]. Furthermore, the fatty acid recoveries obtained by the conventional methods were also compared with one-step direct transesterification methods to select the best lipid and FA extraction method [11]. The efficacy of chloroform/methanol (1/2 and 2/1, *v*/*v*) and dichloromethane/methanol (2/1, *v*/*v*) on lipid extraction was assessed [12]. The accuracy of different lipid extraction methods depends on the solubility of their constituent lipid classes in the solvents employed and the nature of the sample matrix, as both could influence the extent of lipid extraction.

The most commonly encountered solvents in the literature are alkanes (hexane and heptane), alcohols (methanol, ethanol, and propanol), chlorinated (dichloromethane and chloroform), and alternative solvents such as supercritical CO_2_, ionic liquids, and terpenes. The most commonly used analytical method for lipid extraction is chloroform (CHCl_3_) [13]. In other work, it was found that the chloroform/methanol (2:1 *v*/*v*) solvent system showed a higher lipid yield of 28.6% [14]. Factors affecting the extraction process have also been studied [15], revealing that the extraction of microalgae biomass with a moisture content of less than 5%, using a polar and non-polar solvent system, is effective for achieving high lipid yields with a low FFA content, which is suited for conversion into biodiesel.

The resistance of the microalgae cell wall and the different intracellular membranes hampered the lipid extraction process. Various methods of disruption are available to disintegrate these strong cellular walls and membranes and liberate the cell contents [16]. Pre-extraction treatment that breaks down obstacles such as cell walls and membranes will increase the yield of lipids extracted [17,18,19]. To this end, it is essential to ensure that the cell structure is completely disintegrated to increase the efficiency of the extraction process [20,21]. The quantity and quality of functional compounds in the extract depend on the effectiveness of the cell disruption method [22].

For this purpose, different authors have compared different cell disruption procedures on different species of microalgae, such as *Thraustochytrid* strains [23], *Yarrowia lipolytica* [24], *Chlorella vulgaris* [25,26], *Nannochloropsis sp.* [27], *Chlorella*, *Haematococcus* [28], *Botryococus sp.*, *Chlorella vulgaris*, *Scenedesmus sp.* [14], and *Chlorococcum sp.* [29].

Other studies have focused on analysing the impact of specific techniques to facilitate the extraction of different microalgae species. According to previous studies, the quality of biofuel can be improved by modulating elemental and mineral constituents through salt stress while ensuring a net-zero GHG balance [30]. In this sense, we find works analysing the application of mechanical or physical methods such as bead milling [31], high-pressure [32,33], high-speed homogenisation [33], ultrasonication [34,35], microwave treatment [35,36,37,38] pulsed electric field treatment [33,39,40,41], thermolysis (autoclaving) [42], and freeze drying (lyophilisation) [43], as well as non-mechanical methods such as enzymatic cell lysis [44,45] and chemical cell disruption [46,47]. The constituent fatty acids of the extracted fatty matter can be qualitatively and quantitatively determined using chromatographic methods by determining the esters formed after the transesterification process [8].

In this work, starting from *Scenedesmus sp.* as oleaginous biomass, the results obtained when three fatty matter extraction procedures (cold, Soxhlet system, and microwave extraction) were compared. For each of these extraction procedures, different disruption techniques have been employed: ultrasonication, microwave treatment, pulsed electric field treatment, and autoclaving and freeze drying (lyophilisation), with the aim of determining the disruption-extraction technique that would produce the greatest amount of fatty matter, which could be transformed into biodiesel after a transesterification process. The impact of each of these techniques on the composition of the biodiesel in methyl esters was determined by gas chromatography.

## 2. Materials and Methods

### 2.1. Microorganism and Culture Medium

*Scenedesmus sp.* strain BEA 0146B was used. It was provided by the ‘Banco Español de Algas’ (University of Las Palmas de Gran Canaria, Las Palmas de Gran Canaria, Spain), and it was cultivated in the medium Warris-H whose composition was KNO_3_ 100 g/L, MgSO_4_·7 H_2_O 20 g/L, (NH_4_)_2_HPO_4_ 20 g/L, Ca(NO_3_)_2_·4 H_2_O 100 g/L, and HEPES 238.31 g/L. Solution to which 1 mL of each of these solutions was added: micronutrients solution (EDTA C_10_H_14_N_2_Na_2_O_8_·2 H_2_O 3 g/L, H_3_BO_3_ 1.14 g/L, MnCl_2_·4 H_2_O 0.144 g/L, ZnSO_4_·7 H_2_O 0.021 g/L, CoCl_2_·6 H_2_O 0.004 g/L), Fe-EDTA solution (EDTA C_10_H_16_N_2_O_8_ 5.22 g/L, FeSO_4_·7 H_2_O 4.98 g/L, KOH 1N 54.00 mL/L), Vitamins solution (vitamin B_12_ 0.20 mg/L, biotin 1 mg/L, thiamine-HCl 100 mg/L, niacinamide 0.10 mg/L).

The microalga was scaled in a discontinuous stirred photobioreactor of 10 L and maintained at a temperature of 25 °C and a pH of 7.0. Of which the biomass was extracted, and after a centrifugation at 3000 rpm and drying process (at 100 ± 5 °C), the material to be used and weighed 0.4 g for each of the tests.

### 2.2. Cell Disruption

#### 2.2.1. Ultrasonication

Ultrasonication was disrupted using a Branson sonicator (Branson, Dietzenbach, Germany). A quantity of 0.4 g of dried microalgae was used, and 8 mL of distilled water was added to the beaker to maintain the ratio of 1:20. The operating conditions were as follows: 40 KHz of frequency at 50% amplitude and 150 W of power for 1 min.

#### 2.2.2. Autoclaving

This treatment of thermolysis was applied using an Systec autoclave (Systec VB, Linden, Germany). Dried microalgal biomass was introduced in screw-capped tubes with distilled water to maintain the ratio 1:20. The working conditions were 100 °C and 108 kPa for 10 min, in addition to the heating period (at a rate of 7.0 °C/min) and cooling period (at 0.15 °C/min) of the equipment.

#### 2.2.3. Microwaves

For microwave pretreatment, a Milestone microwave oven (Ethos One, Milestone, Sorisole, Italy) was used. In each of the 10 microwave reactors, 0.5 g of dry biomass was introduced into 10 mL of distilled H_2_O, maintaining a solid-liquid ratio of 1:20. As many reactors are used, as much microalgal biomass needs to be treated. The operating conditions to carry out the disruption applied for 5 min were as follows: 100 °C temperature, 20 bar pressure, and 500 W power, with stirring at 50%.

#### 2.2.4. Pulsed Electric Field Treatment

The electrolysis equipment from Alonso Alegre and Cia (Bilbao, Spain). was used. Dry microalgal biomass (2.5 g) was mixed with 50 mL of tap water in a beaker to contain all electrolytes required to promote electron conversion. A solid: liquid ratio of 1:20 was maintained.

The platinum mesh electrodes (electrodes according to Fischer made of Platinum/Iridium 90/10%, EL03/2, Heraeus Platinum Labware, Pforzheim, Germany) and the glass stirring rod were attached to the process equipment. Once the assembly is completed, the sample is subjected to a value of 10 V and 10 A of intensity, although the applied value is perceived not to exceed 3 A. Foams are formed during pretreatment, and the sample is heated. The process stops when these effects are no longer directly observed and the amperage value decreases. The treatment time will depend on the amount of biomass-water mixture introduced into the electrodes; in our case, it was 10 min.

Once the wet biomass has been pretreated in a sonicator Branson, (Dietzenbach, Germany), microwave (Ethos One, Milestone, Sorisole, Italy), autoclave (Systec VB, Linden, Germany), and pulsed electric field equipment (Alonso Alegre and Cia, Bilbao, Spain), it is placed in a convection oven (Selecta, Abrera, Barcelona, Spain) at 40 °C to remove all moisture and stored in a cool, dry place until further use.

#### 2.2.5. Freeze Drying (Lyophilisation)

The freeze-drying disruption was carried out in a lyophilisator (Termo Savant, Wien, Austria) at −50 °C, and the pressure range was 30 μbar to 50 mbar. The same solid: liquid and lyophilised biomass ratio was maintained; no further drying was required. The product was stored in a dry and cool place until use.

### 2.3. Extraction

Dry pretreated biomass was subjected to three different fat-extraction procedures: hot, cold, and microwave extraction.

#### 2.3.1. Hot Extraction in Soxhlet

For this type of extraction, the amount of biomass to be extracted is placed inside an extraction cartridge in the Soxhlet extractor body. In a previously weighed flask, 100 mL of the extraction solvent mixture chloroform/methanol is added in the proportion 2:1 *v*:*v* [34], keeping the system in recirculation for 15 h to ensure total fat matter separation.

After the extraction time, the solvent mixture is separated in a Heidolph rotary evaporator. The amount of lipid removed is determined using Equation (1):

(1) $$\%\;\mathrm{Lipids}\;=\;\frac{M_{L}}{M}\times 100$$

where *M_L_* is the lipid weight in grams, and *M* is the dry biomass weight in grams [48].

#### 2.3.2. Cold Extraction

This type of extraction is performed using a magnetic stirrer (Selecta, Agimatic-E, Barcelona, Spain) The dry biomass to be extracted is introduced into an Erlenmeyer flask, previously filled with the chloroform-methanol solvent mixture at a ratio of 2:1 (*v*:*v*). For 15 h, the mixture is kept agitated with magnetic spin at 500 rpm without switching, connected to a coolant to prevent evaporation at a temperature below 25 °C.

A first centrifugation step (10 min at 2500 rpm) is used to separate the solid and liquid fractions, followed by a rotary evaporator (Heidolph, Schwabach, Germany) step in order to separate the solvent from the extracted lipids. The % lipid obtained is determined by Equation (1).

#### 2.3.3. Microwave Extraction

Microwaves (Ethos One, Milestone, Sorisole, Italy) were used for extraction and disruption. Microwave extraction was developed in the Ethos One microwave oven using the solvent mixture chloroform-methanol at the proportion 2:1 (*v*/*v*). The dried microalgal biomass and the solvent mixture were introduced into the microwave reactors, and the microwave extraction process was performed under the following conditions: 500 w, 100 °C, stirring at 50%, 20 bar of pressure, and 30 min of treatment. The temperature was controlled using a thermocouple.

The liquid fraction was separated from the extracted biomass, and the first centrifugation step (10 min) was performed. Then, the liquid fraction was added to pre-weighed flasks for use in the Heidolph rotary evaporator. The gravimetric lipid percentage was calculated using Equation (1).

### 2.4. Transesterification

The extracted lipid fraction was transesterified. This reaction occurs between the lipid fraction’s triglyceride constituents (in a proportion greater than 90% by weight) and a short-chain alcohol, such as methanol. The theoretical molar alcohol-oil ratio is 6:1, although a higher ratio is used for total reagent transformation, and a catalyst is required. The product of this reaction will be a mixture of biodiesel [49] and glycerine to be separated.

Once the separated lipid fraction has been gravimetrically quantified, the transesterification reaction is performed in an orbital stirrer (Infors HT Ecotron, Bottmingen, Switzerland). 0.05 g of KOH was used together with 10 mL of methanol, which was added directly to the flasks containing the lipids and subjected to orbital stirring for 20 min at 60 °C and 100 rpm. After transesterification, FAME is separated from glycerin by decantation. The amount of esters is determined using Equation (2) as follows:

(2) $$\%\;\mathrm{Esters}\;=\;\frac{M_{E}}{M}\times 100$$

where *M_E_* is the ester’s weight in grams, and *M* is the dry biomass weight in grams.

### 2.5. Determination of Fatty Acids by GC

The organic phase was separated and washed with n-hexane to determine the FAME profile by gas chromatography.

A gas chromatograph (Shimadzu GC-2020 Plus with autosampler AOC-20i+s with FID detector, Duisburg, Germany) was used. A BP20 column of 30 m in length with an inner diameter of 0.25 mm was used at 250 °C, with H_2_ as the carrier gas at a pressure of 100 kPa and a total flow of 250 mL/min. The temperature of the FID detector was set to 280 °C, and the injection mode was split with a ratio of 100.

A standard mixture of ME 76 (100 mg) of Larodan with 10 methyl esters was used to determine the fatty acid profile, from which the calibration curve of the method used was prepared Its composition was C16:0 methyl palmitate 4%, C18:0 methylstearate 1%, C18:1 methyl oleate 58%, C18:2 methyl linoleate 20%, C18:3 methyl linolenate 10%, C20:0 methyl eicosanoate (araquidic) 1%, C20:1 methyl eicosenoate 2%, C22:0 methyl behenate 1%, C22:1 methyl erucate 2%, and C24:0 methyl lignocerate 1%. The quantity of fatty acids obtained shall be expressed in relative %, out of the 10 methyl esters analysed as the most frequently found in microalgal biomass [50].

### 2.6. SEM Analysis

**Figure 1:**
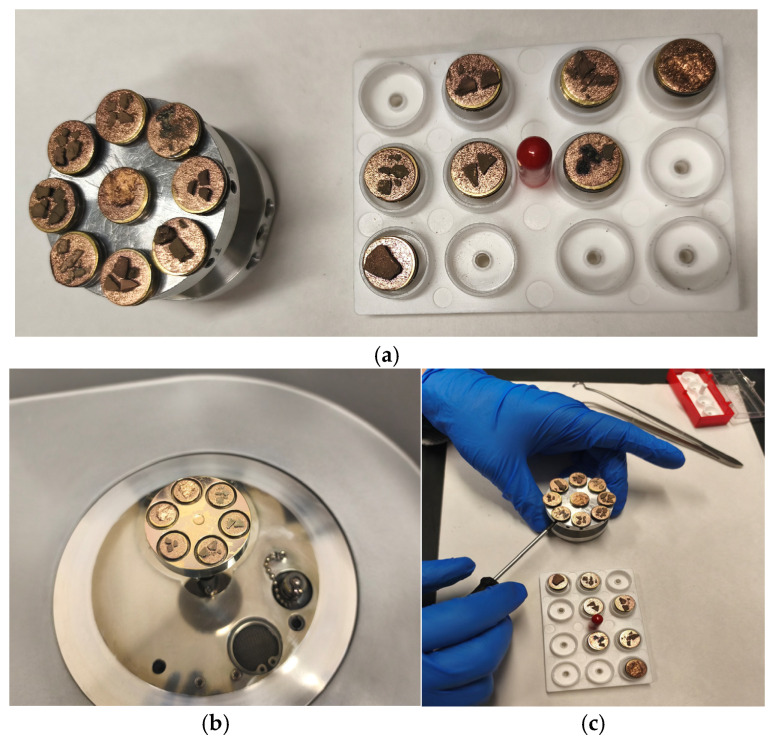
Stages of the metallisation process: (**a**) assembly, (**b**) gold leaf coating, and (**c**) removal of the pedestals.

A topographical analysis of the samples was carried out using scanning electron microscopy (SEM) on a high-resolution Merlin microscope (Carl Zeiss, Dublin, CA, USA). The samples were coated with gold after being mounted on 12.7 mm pedestals using a combined high-vacuum metallisation and evaporation system, (Quorum Q150T ES, Laughton, UK), which enabled a thin, conductive gold film to be deposited on the sample surface for observation under the SEM microscope (Figure 1). The samples analysed by SEM were dried at 40 °C after undergoing the disruption procedures.

## 3. Results and Discussion

After applying the considered disrupting treatments, both to the non-lyophilised microalgal biomass (wet biomass) and to the lyophilised biomass, the results in relation to the % of lipids obtained from the extraction process (considering the three extraction procedures used), as well as the percentage of esters obtained after the transesterification process and the profile of fatty acids obtained, has been compared to determine the impact that these methods of disruption and extraction on the biomass of *Scenedesmus sp.*

### 3.1. Wet Scenedesmus sp.

The initial lipid content of *Scenedesmus sp.* ranges from 19.6% to 21.1% [49]. The efficiency of lipid extraction depends on the microalgae species and pretreatment methods [14].

#### 3.1.1. Lipids and Esters

Using microalgae biomass from *Scenedesmus sp.* without prior lyophilisation (wet *Scenedesmus sp.* biomass), three extraction procedures were applied: cold (at a temperature below 25 °C), in Soxhlet (hot), and using microwaves for extraction to separate the lipid fraction from the initial dry biomass to samples that have been disrupted by pulsed electric field (EP), ultrasounds (US), microwaves (MW), autoclaving (AC), and lyophilisation (LYO) and compare the results reached. In addition to the sample that has not been subjected to any disruption process (WD). The percentage of esters obtained after transesterification was also analysed.

**Figure 2:**
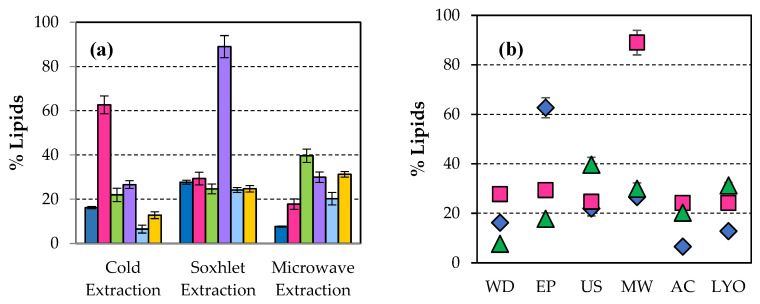
(**a**) Percentage of lipids extracted applying different procedures of extraction to disrupted biomass of *Scenedesmus sp.*: 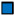 Without disruption (WD), 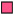 Pulsed electrid field (EP), 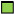 Ultrasonication (US), 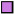 Microwaves (MW), 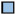 Autoclaving (AC), and 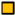 Lyophilisation (LYO). (**b**) Percentage of lipids extracted from the disrupted biomass of *Scenedesmus sp.* applying different procedures of extraction: 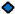 in cold, 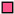 Soxhlet system, and 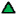 microwave oven.

Figure 2a shows that the percentage of lipids extracted by the Soxhlet system was significantly higher when the original biomass was not pre-processed in the microwave (88.97). This value represents an increase of 81.77% compared with 16.22% of lipids in the unpretreated WD. The best result for separated lipids was observed when the biomass of *Scenedesmus sp.* was subjected to a pulsed electric field (62.68%) when the lipid extraction was performed cold. This represents an increase of 53.21% in the raw material content. When extraction was developed in a microwave, the highest value in the extracted lipids was reached when the original biomass was pretreated with ultrasound, 39.65%, which is an increase of 80.63% in the initial lipid content of WD biomass. In Figure 2, it is possible to visualise the highest percentage of lipids using microwaves as a disruption when the extraction was carried out hot (Soxhlet system), while with the pulsed electric field technique, better results are obtained by extracting cold. In another study comparing ultrasonication, microwave, electroporation, and autoclave, it was found that using microwaves is the best procedure for an incremental % of lipid extraction, although this figure was only 38% [51]. Microwaving with *Nannochloropsi* also gave better results in lipid production than ultrasonication [52]. With *Scenedesmus sp.*, Halim et al. also found that the application of microwaves gave a better % of lipid yield [29]. Microwaves are electromagnetic radiation with frequencies ranging from 0.3 to 300 GHz. Microwave-assisted heating uses a noncontact heat source that can penetrate biomaterials, interact with polar molecules like water in the biomass and uniformly heat the whole sample. The main advantages of microwave-assisted extraction are higher oil yield, superior quality, and reduced extraction time [53].

**Figure 3:**
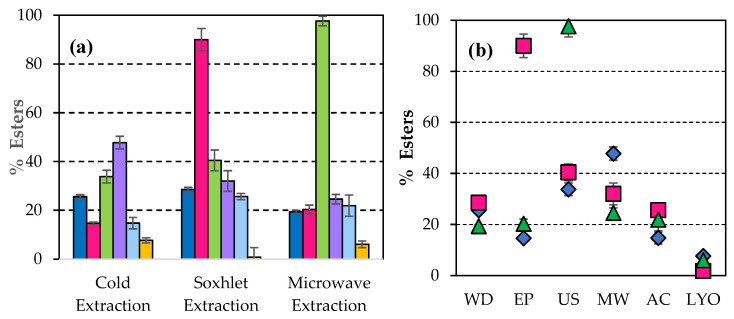
(**a**) Percentage of esters separated after transesterification of lipids extracted from disrupted biomass of *Scenedesmus sp.* with the methods: 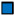 Without disruption (WD), 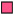 Pulsed electric field (EP), 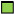 Ultrasonication (US), 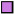 Microwaves (MW), 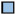 Autoclaving (AC), and 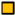 Lyophilisation (LYO). (**b**) Percentage of esters separated after transesterification of lipids when the biomass of *Scenedesmus sp.* was disrupted and extracted applying the procedures of extraction: 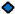 in cold, 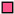 Soxhlet system, and 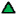 microwave oven.

After the transesterification of the extracted lipids to the previously disrupted biomass, the greatest amount of esters (Figure 3a) was obtained when the extraction of lipids was carried out in microwaves, 97.64% (referred to as raw microalgal biomass dry weight) having previously applied to the untreated biomass, an ultrasonic pretreatment, representing an increase of 70.80% in the ester content of WD biomass (28.51%). Comparing different extraction methods, sonication improved both lipid and ester production percentages [12]. The sample pretreated with microwaves showed a higher ester percentage after cold extraction.

Figure 3b shows the highest percentage of esters obtained after ultrasonication with microwave extraction, followed by the value achieved with pulsed electric field disruption when the sample is extracted in hot water.

When lyophilisation was applied to the original biomass as a disruption technique, the best result in lipid percentage was achieved when extraction was carried out in a microwave oven (Figure 2). This treatment did not produce any significant effect in the percentage of esters (Figure 3).

#### 3.1.2. FAME Determination

After the transesterification process, the percentage of FAME is expressed with respect to the 10 methyl esters considered in the GC analysis. The methyl ester proportion corresponds to the fatty acid from which the ester is derived. Therefore, the fatty acid profile for each analysed sample in the considered methyl ester range can be determined.

The compounds between C16 and C24 have been studied with regard to the profile of fatty acids produced, among which are the most abundant fatty acids present in the microalgal biomass. These include palmitic acid (hexadecanoic, or C16:0), stearic acid (octadecanoic, or C18:0), oleic acid (9-octadecenoic, C18:1), linoleic acid (9,12-octadecadienoic, C18:2), linolenic acid (9,12,15-octadecatrienoic, C18:3), eicosanate acid (arachidic acid C20:0), eicosenoate acid (C20:1), behenic acid (docosanoic C:22:0), erucic acid (cis-13 docosenoic acid or C22:1), and up to lignoceric acid (C24). Methyl esters were determined by gas chromatography after the extracted lipids were transesterified.

**Figure 4:**
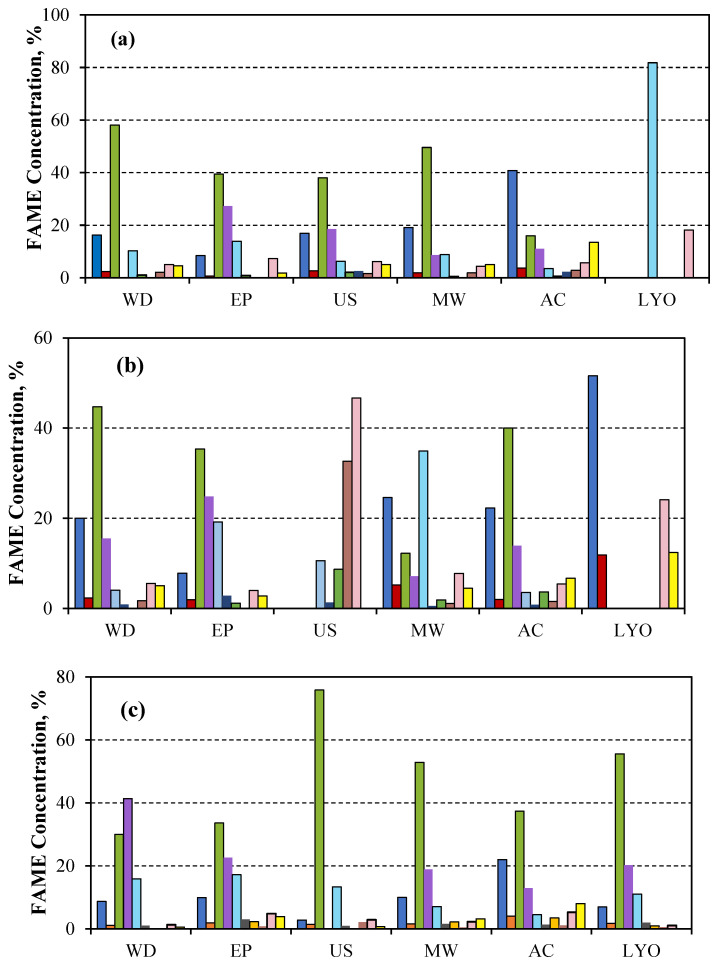
Variation of % FAME reached after transesterification when lipids of *Scenedesmus sp.* biomass were extracted in (**a**) cold, (**b**) Soxhlet system, and (**c**) microwave oven and disrupted with different methods: Without disruption (WD), Pulsed electric field (EP), Ultrasonication (US), Microwaves (MW), and Autoclaving (AC). 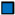 Methyl palmitate, 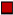 Methyl stearate, 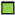 Methyl oleate, 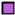 Methyl linoleate, 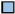 Methyl linolenate, 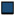 Methyl eicosanoate, 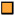 Methyl eicosenoate, 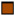 Methyl benehate, 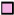 Methyl erucate, and 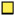 Methyl lignocerate.

In Figure 4a, when the biomass extracted from *Scenedesmus sp.* was extracted cold, oleic acid was found to be the most abundant, followed by palmitic acid, whose percentage increased when the biomass was autoclaved. A greater predominance of linolenic acid and erucic acid was observed only when the biomass was subjected to a lyophilisation pretreatment; the other acids were not detected in this case.

When extraction was performed in the Soxhlet system (Figure 4b), the oleic acid predominance changed to a higher palmitic acid abundance when the biomass of *Scenedesmus* was previously lyophilised. The application of ultrasounds increased erucic acid, whereas microwave-disrupting treatment increased linolenic acid.

During microwave extraction (Figure 4c), a decrease in the proportion of linoleic acid (C18:2), a polyunsaturated fatty acid, was observed. Conversely, the proportion of oleic acid (C18:1), a monounsaturated fatty acid, increased following the application of the different disruption methods; this effect was most pronounced when ultrasound was applied. This results in the production of a higher quality biodiesel due to the increase in oxidative stability [54]. In the same way, a lower proportion of long-chain fatty acids was also observed, which allows for the production of a lower-viscosity biodiesel, thereby enhancing its quality [55].

By extracting lipids from *Chlorococcum sp.*, oleic acid C18:1 was found to be predominant [9]. In addition, *Nostoc sp.* and *Tolypothrix sp.* showed higher oleic acid content when sonication was applied, when the cells were disrupted using the sonication method [56].

### 3.2. Lyophilised Scenedesmus sp.

When microalgae biomass from *Scenedesmus sp.* lyophilised previously, the three extraction procedures were applied: cold, in Soxhlet system (hot) and using microwaves for extraction, in order to separate the lipid fraction from the initial dry biomass to samples that have been subjected to processes of disruption by pulsed electric field (EP), ultrasounds (US), microwaves (MW), and autoclaving (AC). In addition to the sample that has not been subjected to any disruption process (WD). The percentage of esters obtained after the transesterification of the lyophilised microalgal biomass was also compared.

#### 3.2.1. Lipids and Esters

The freeze-drying (lyophilisation) process was applied as a conservation technique for the microalgal biomass. In this study, we analysed the effect of extraction processes and other disruption techniques on previously lyophilised microalgal biomass.

**Figure 5:**
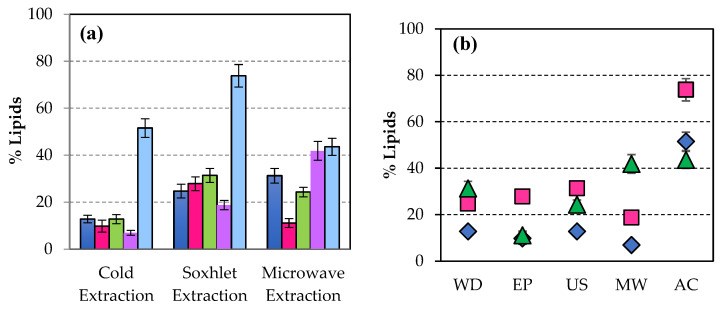
(**a**) Percentage of lipids extracted applying different procedures of extraction to disrupted lyophilised biomass of *Scenedesmus sp.*: 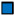 Without disruption (WD), 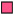 Pulsed electric field (EP), 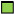 Ultrasonication (US), 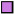 Microwaves (MW), and 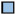 Autoclaving (AC). (**b**) Percentage of lipids extracted from disrupted lyophilised biomass of *Scenedesmus sp.* applying different procedures of extraction: 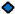 in cold, 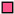 Soxhlet system, and 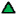 microwave oven.

**Figure 6:**
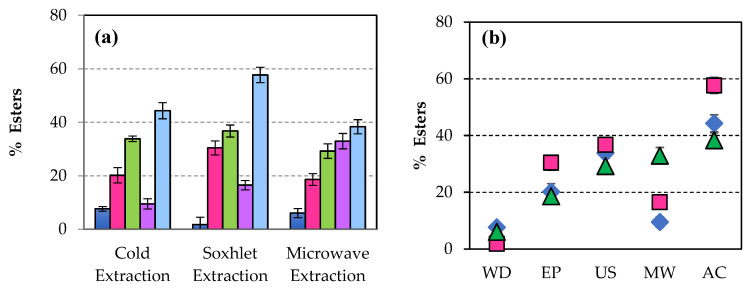
(**a**) Percentage of esters separated after transesterification of lipids extracted from disrupted lyophilised biomass of *Scenedesmus sp.* with the methods: 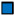 Without disruption (WD), 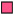 Pulsed electric field (EP), 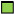 Ultrasonication (US), 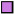 Microwaves (MW), and 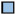 Autoclaving (AC). (**b**) Percentage of esters separated after transesterification of lipids when the lyophilised biomass of *Scenedesmus sp.* was disrupted and extracted applying the procedures of extraction: 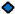 in cold, 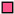 Soxhlet system, and 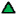 microwave oven.

However, when lyophilisation was applied to the original biomass, and on this lyophilised biomass, other disruption techniques were applied, the best results after extraction processes were achieved when disruption was carried out in an autoclave. The lipid percentage was significantly higher when extraction was performed in Soxhlet (73.78%), indicating an increase of 66.45% with respect to freeze-dried (lyophilised) biomass without additional disruption. In addition, after extraction in cold (51.54%) and microwave ovens (43.57%), the best result for the disruption technique applied to freeze-dried microalgal biomass, compared to the percentage of lipids extracted, was obtained by the autoclave treatment. Achieving the highest increase in lipid percentage of biomass of *Scenedesmus sp.* liophilised, no additional disrupting treatment for cold-extracted and autoclaved samples, 75.15% (Figure 5a). Figure 5b shows that the best results are obtained for samples subjected to autoclaving compared with those obtained for other treatments. An equivalent behaviour was observed with respect to ester production (Figure 6).

Applying-freeze drying to *Scenedesmus sp.*, followed by lipid extraction, a lipid yield of 29.65% was achieved [57].

#### 3.2.2. FAME Determination

When biomass from lyophilised *Scenedesmus sp.* has been used. After the transesterification process, the percentage of FAME is expressed with respect to the 10 methyl esters considered in the GC analysis.

**Figure 7:**
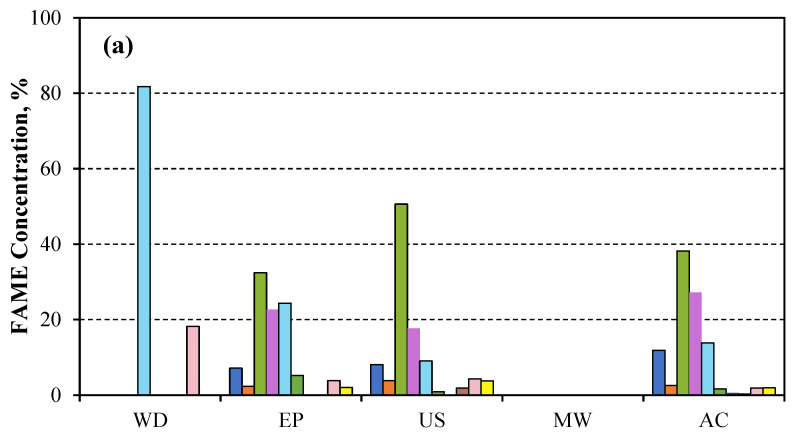

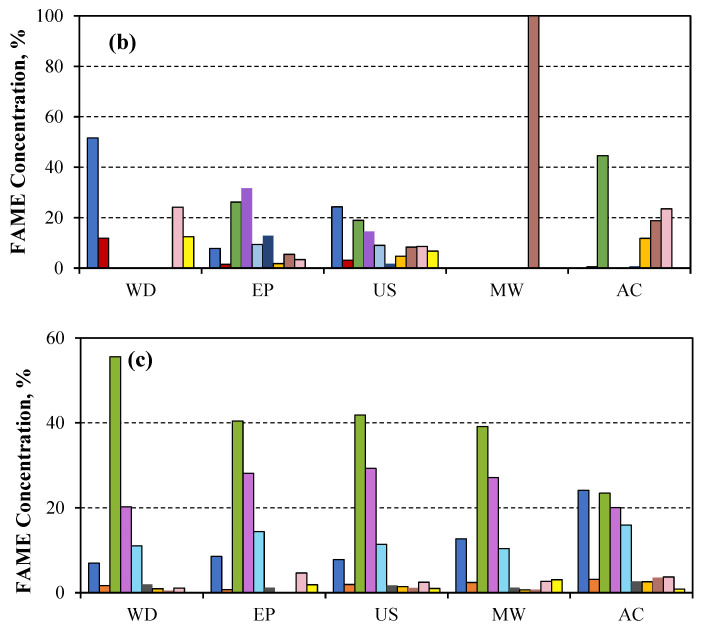
Variation of % FAME reached after transesterification when lipids of *Scenedesmus sp.* lyophilised biomass were extracted in (**a**) in cold, (**b**) in Soxhlet, and (**c**) in microwave oven and disrupted with different methods: Without disruption (WD), Pulsed electric field (EP), Ultrasonication (US), Microwaves (MW), and Autoclaving (AC). 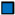 Methyl palmitate, 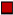 Methyl stearate, 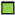 Methyl oleate, 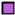 Methyl linoleate, 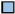 Methyl linolenate, 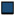 Methyl eicosanoate, 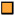 Methyl eicosenoate, 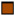 Methyl benehate, 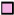 Methyl erucate, and 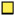 Methyl lignocerate.

The application of disruption procedures to previously lyophilised biomass, when the lipids were cold extracted (Figure 7a), led to an increase in oleic acid when ultrasonic pretreatment was applied. This effect was less when autoclaving was used and even less when the pulsed electric field technique was used.

When the previously lyophilised biomass of *Scenedesmus sp.* was extracted in a Soxhlet system (Figure 7b), the only acid determined when microwaves were applied as a disruption procedure was behenic acid.

Microwave extraction of the previously lyophilised biomass allowed for an increase in the proportion of linoleic acid (C18:2) when ultrasonic, electroporation, and microwave pretreatments were applied as disruption methods (Figure 7c). This coincides with observations made in the case of *Chlorella protothecoides*, in which the lyophilisation improved efficient lipid extraction from microalgal biomass, with linoleic acid (C18:2) being the most abundant [21].

#### 3.2.3. SEM Analysis

**Figure 8:**
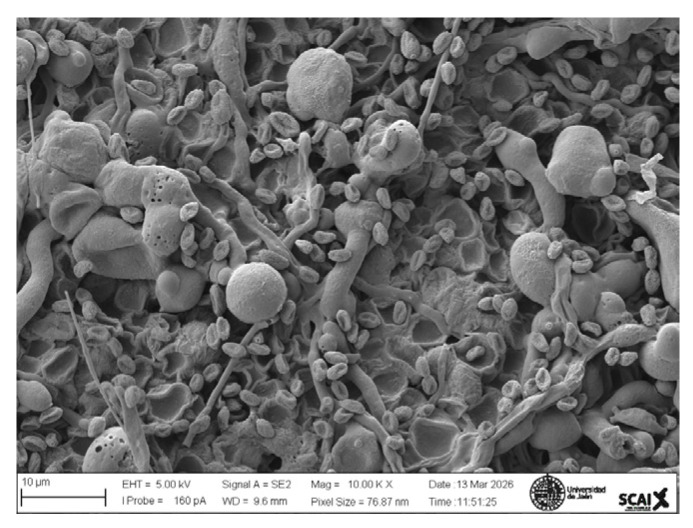
Initial untreated biomass *Scenedesmus sp*.

Table 1 shows SEM images of wet *Scenedesmus sp.* samples, taken after disruption and extraction, compared with the image of the untreated biomass (Figure 8). The images were obtained under the same conditions of voltage (5.00 kV) and magnification (10,000×).

The images show the effect on the surface of all disruption techniques applied to wet *Scenedesmus sp.* following extraction. Greater surface damage appears to be observed when samples have been pretreated with ultrasound and microwaves and extracted using the Soxhlet system and microwaves, compared with other disruption and extraction procedures. This greater surface damage could explain the results obtained regarding lipid extraction yield and the predominance of oleic acid over other fatty acids.

## 4. Conclusions

This study concludes that the efficiency of lipid recovery and the resulting biodiesel quality from *Scenedesmus sp.* are governed by the specific interaction between cell disruption and extraction methods, where microwave disruption combined with Soxhlet extraction achieves the highest total lipid yield. While microwave disruption paired with Soxhlet extraction maximises quantity, ultrasonic disruption integrated with microwave extraction is most effective for maximising both total lipid and methyl ester production, whereas autoclaving is the superior disruption technique for freeze-dried biomass. Furthermore, the FAME profile can be strategically manipulated to enhance biodiesel quality, as oleic acid production is maximised through ultrasonic disruption with microwave extraction, palmitic acid through autoclaving with cold extraction, and linolenic or erucic acids by applying microwave or ultrasonic disruption followed by Soxhlet extraction. Ultimately, these findings indicate that the selection of methodologies involves a critical trade-off between energy conservation through cold extraction and time efficiency via microwave-assisted processes, allowing for the implementation of optimised protocols tailored to produce biodiesel with specific chemical characteristics and high-performance fatty acid profiles. It is worth noting that future development of this work would involve developing the best treatments (ultrasonic disruption with microwave extraction) at mini-plant and pilot-plant scales, with the ultimate goal of testing the biodiesel obtained in a diesel engine located on a test bench.

## Figures and Tables

**Table 1:** SEM images of wet *Scenedesmus sp.* samples subjected to disruption procedures.

	Cold Extraction	Soxhlet Extraction	Microwave Extraction
**Without disruption**	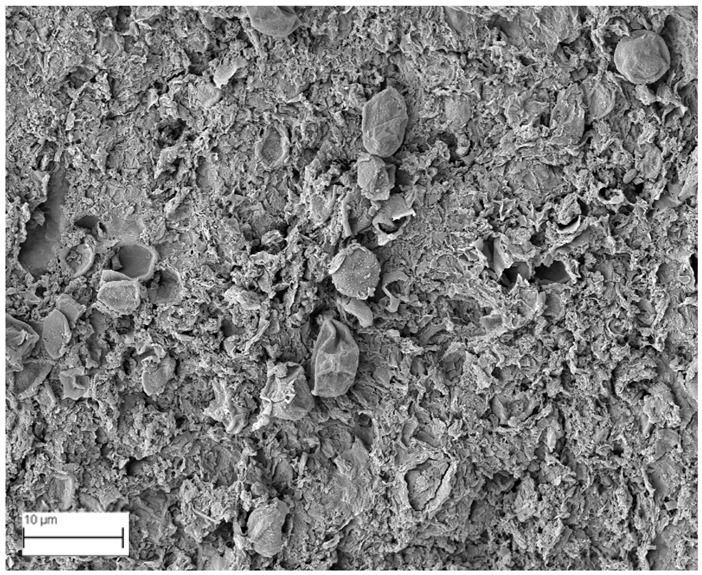	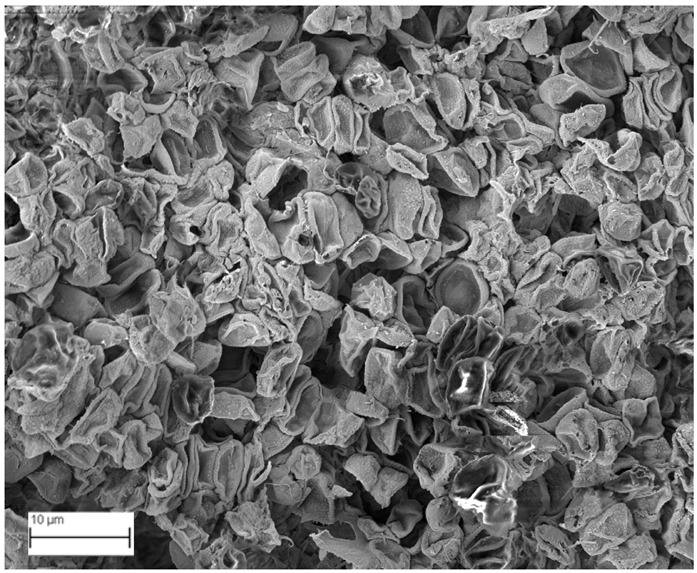	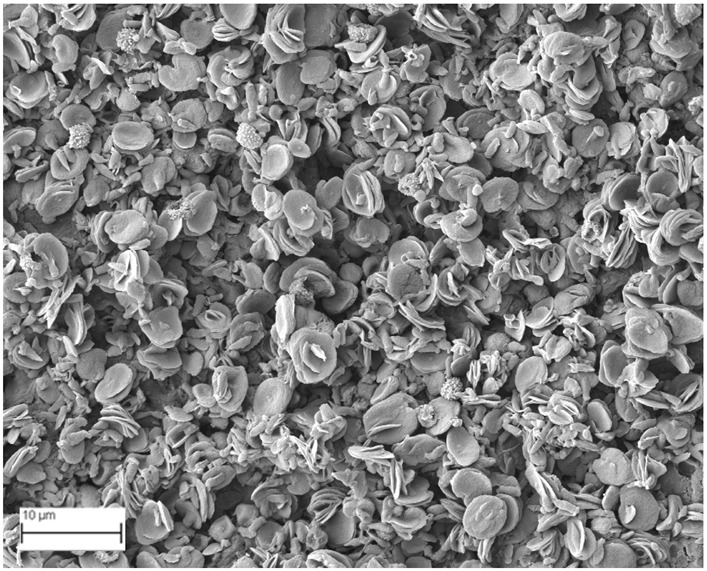
**Pulsed electric field**	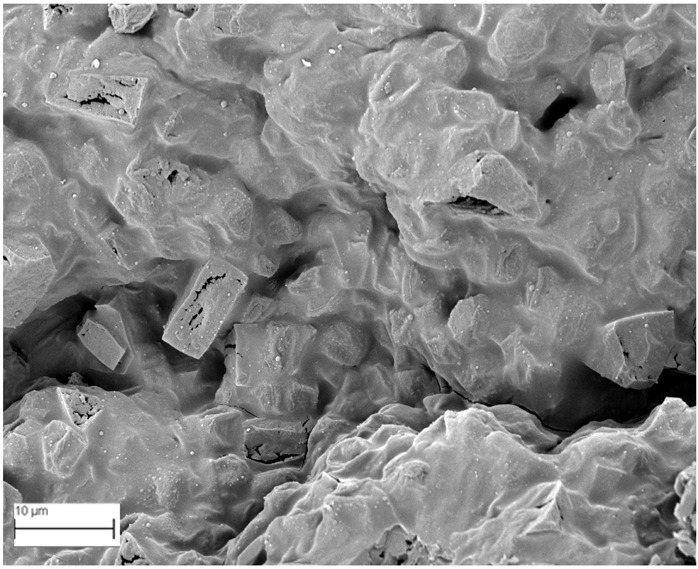	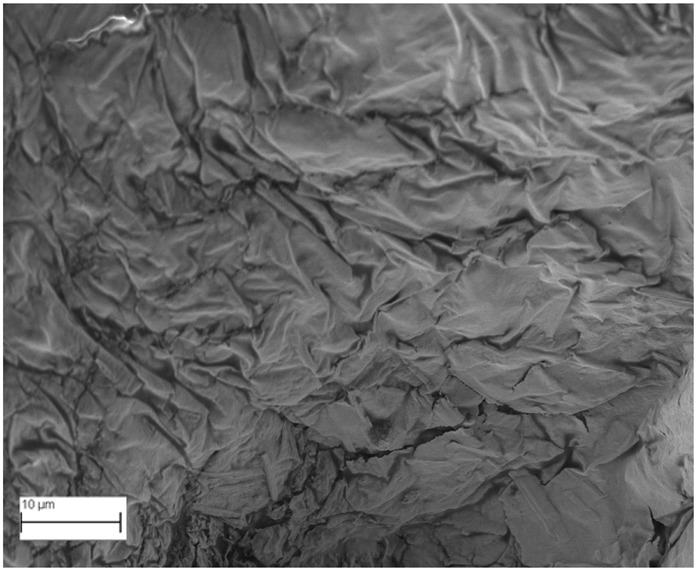	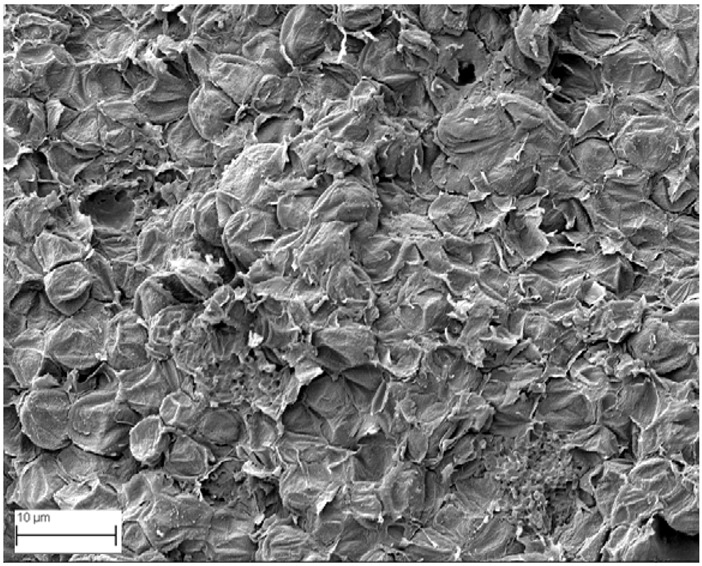
**Ultrasonication**	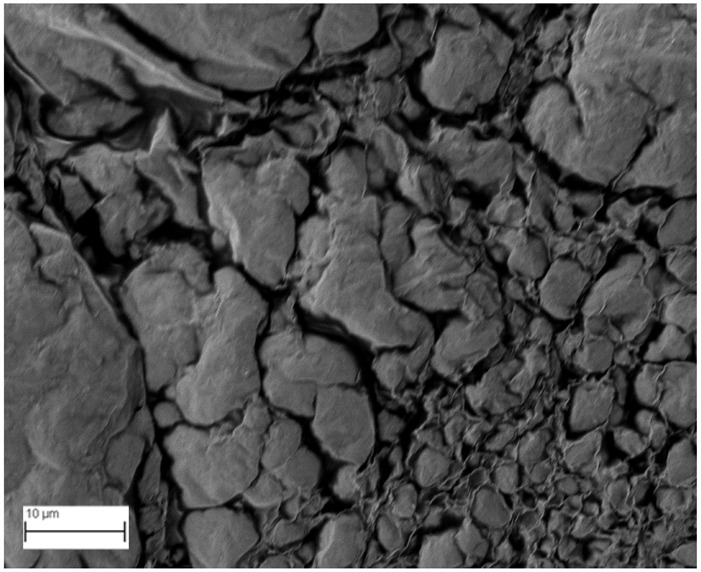	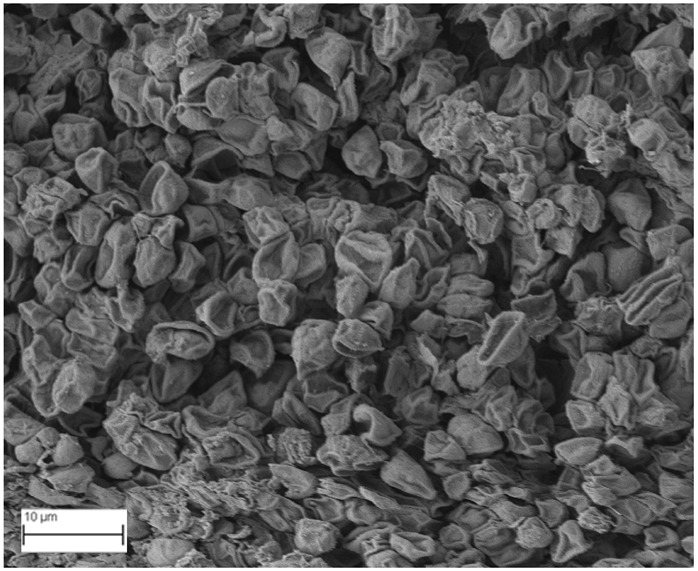	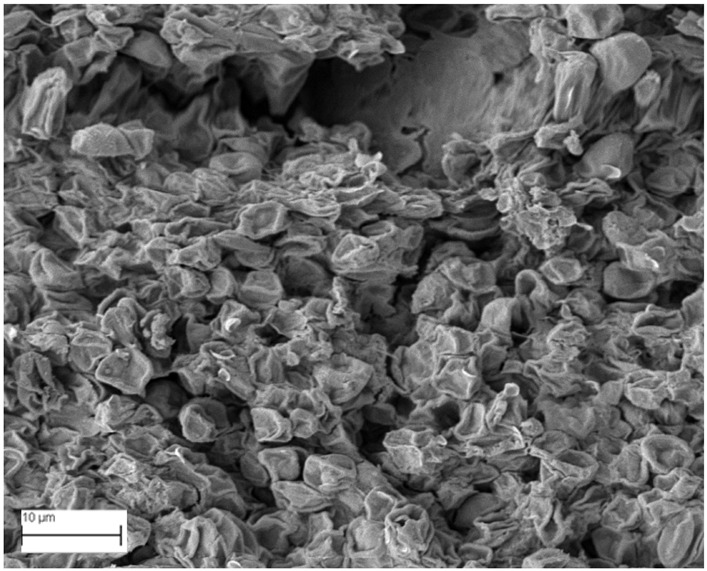
**Microwaves**	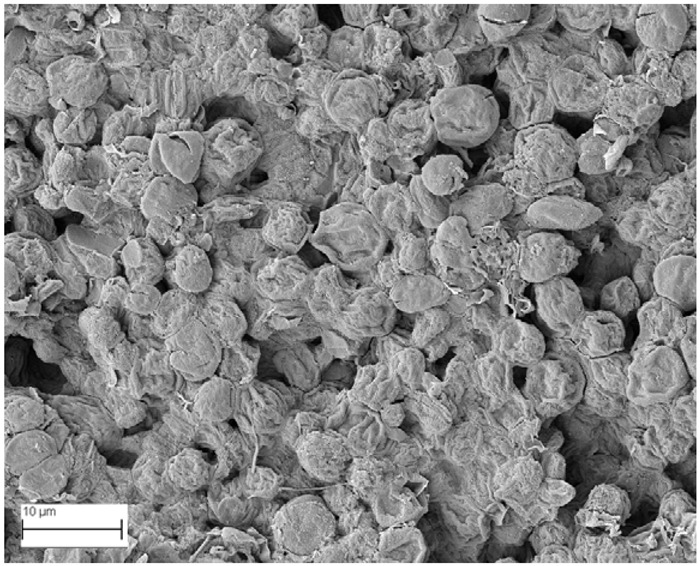	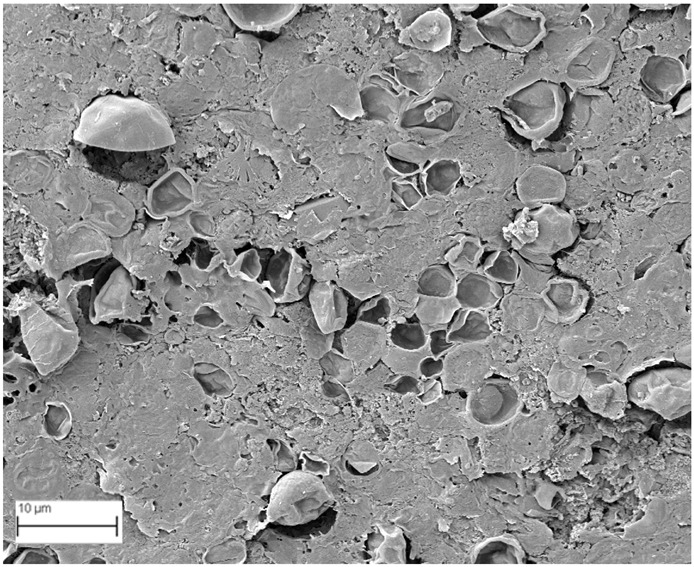	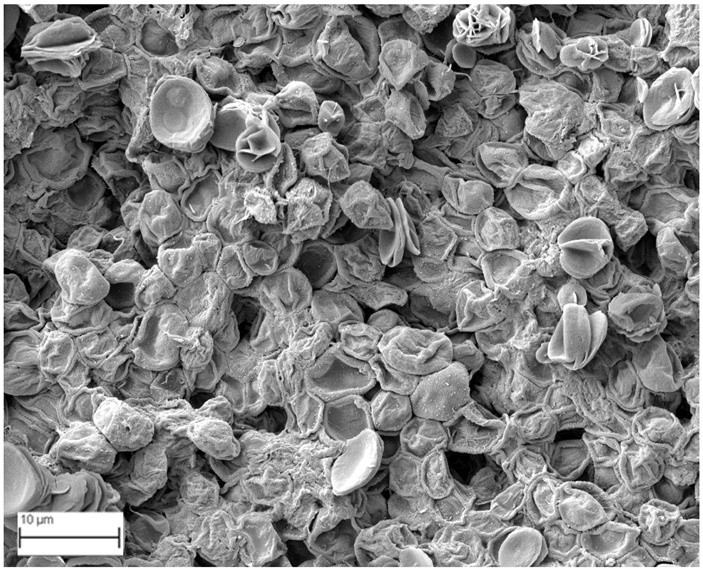
**Autoclaving**	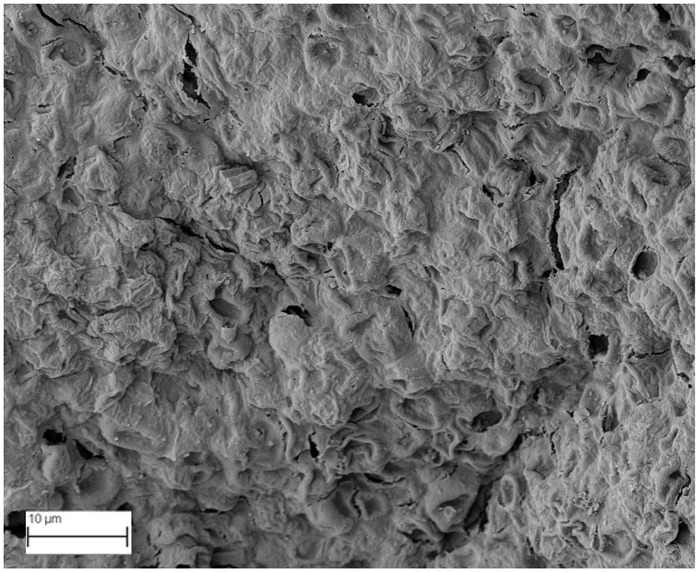	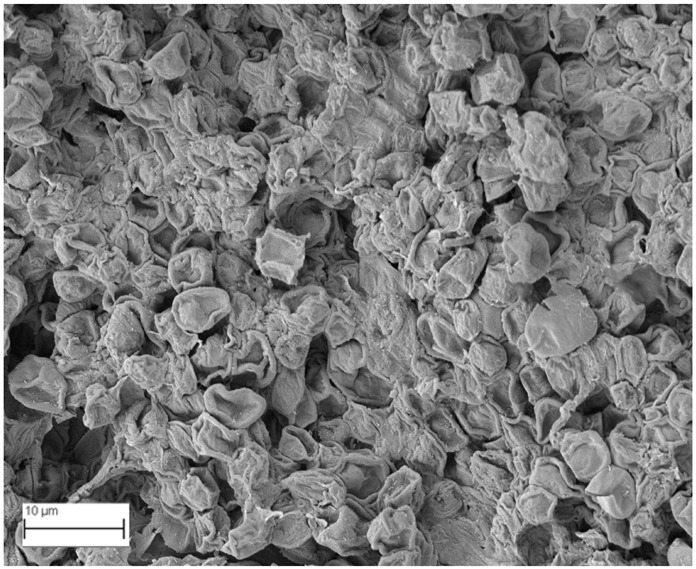	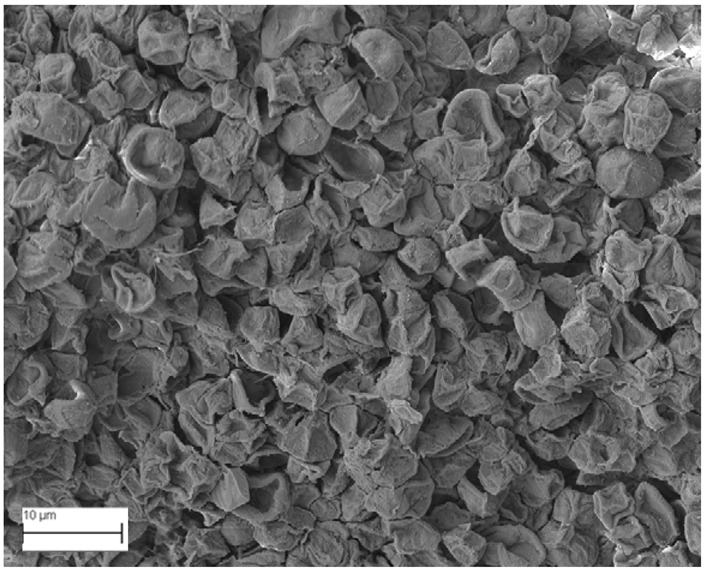

## Data Availability

The original contributions presented in this study are included in the article. Further inquiries can be directed to the corresponding author.

## References

[1] Amaro H.M., Guedes A.C., Malcata F.X. (2011). Advances and Perspectives in Using Microalgae to Produce Biodiesel. Appl. Energy.

[2] Huang G.H., Chen F., Wei D., Zhang X.W., Chen G. (2010). Biodiesel Production by Microalgal Biotechnology. Appl. Energy.

[3] Irena W.B., UNSD, WHO, IEA (2021). The Energy Progress Report 2022.

[4] European Commission (2019). Going Climate-Neutral by 2050—A Strategic Long-Term Vision for a Prosperous, Modern, Competitive and Climate-Neutral EU Economy.

[5] Teo C.L., Idris A. (2014). Enhancing the Various Solvent Extraction Method via Microwave Irradiation for Extraction of Lipids from Marine Microalgae in Biodiesel Production. Bioresour. Technol..

[6] Torres S., Acien G., García-Cuadra F., Navia R. (2017). Direct Transesterification of Microalgae Biomass and Biodiesel Refining with Vacuum Distillation. Algal Res..

[7] Kim B., Young H., Son J., Yang J., Chang Y., Lee J.H., Lee J.W. (2019). Simplifying Biodiesel Production from Microalgae via Wet in Situ Transesterification: A Review in Current Research and Future Prospects. Algal Res..

[8] Christie W.W., The Scottish Crop Research Institute (1993). Preparation of Ester Derivatives of Fatty Acids for Chromatographic Analysis. Advances in Lipid Methodology—Two.

[9] Halim R., Danquah M.K., Webley P.A. (2012). Extraction of Oil from Microalgae for Biodiesel Production: A Review. Biotechnol. Adv..

[10] Crocker M. (2014). Lipid Extraction from *Scenedesmus sp.* Microalgae for Biodiesel Production Using Hot Compressed Hexane. Fuel.

[11] Chen C., Huang C., Ho K., Hsiao P., Wu M. (2015). Biodiesel Production from Wet Microalgae Feedstock Using Sequential Wet Extraction/Transesterification and Direct Transesterification Processes. Bioresour. Technol..

[12] Kumari P., Reddy C.R.K., Jha B. (2011). Comparative Evaluation and Selection of a Method for Lipid and Fatty Acid Extraction from Macroalgae. Anal. Biochem..

[13] Angles E., Jaouen P., Pruvost J., Marchal L. (2017). Wet Lipid Extraction from the Microalga *Nannochloropsis sp.*: Disruption, Physiological Effects and Solvent Screening. Algal Res..

[14] Lee J., Yoo C., Jun S., Ahn C., Oh H. (2010). Comparison of Several Methods for Effective Lipid Extraction from Microalgae. Bioresour. Technol..

[15] Kumar R., Thai T., Doan Y., Philip J. (2013). Factors Affecting Cellular Lipid Extraction from Marine Microalgae. Chem. Eng. J..

[16] Shivakumar S., Serlini N., Esteves S.M., Miros S., Halim R. (2024). Cell Walls of Lipid-Rich Microalgae: A Comprehensive Review on Characterisation, Ultrastructure, and Enzymatic Disruption. Fermentation.

[17] Steriti A., Rossi R., Concas A., Cao G. (2014). A Novel Cell Disruption Technique to Enhance Lipid Extraction from Microalgae. Bioresour. Technol..

[18] Show K., Lee D., Tay J., Lee T., Chang J. (2015). Microalgal Drying and Cell Disruption—Recent Advances. Bioresour. Technol..

[19] Nee W., Loke P., Chuan T., Ching J., Ng E., Chang J. (2018). Mild Cell Disruption Methods for Bio-Functional Proteins Recovery from Microalgae—Recent Developments and Future Perspectives. Algal Res..

[20] Howlader S., Rai N., Todd W. (2018). Improving the Lipid Recovery from Wet Oleaginous Microorganisms Using Different Pretreatment Techniques. Bioresour. Technol..

[21] Piasecka A., Krzemiñska I., Tys J. (2014). Physical Methods of Microalgal Biomass Pretreatment. Int. Agrophysics.

[22] Rakesh S., Dhar D.W., Prasanna R., Saxena A.K., Saha S., Shukla M., Sharma K. (2015). Cell Disruption Methods for Improving Lipid Extraction Efficiency in Unicellular Microalgae. Eng. Life Sci..

[23] Byreddy A.R., Gupta A., Barrow C.J., Puri M. (2015). Comparison of Cell Disruption Methods for Improving Lipid Extraction from Thraustochytrid Strains. Mar. Drugs.

[24] Meullemiestre A., Breil C., Abert-vian M., Chemat F. (2016). Microwave, Ultrasound, Thermal Treatments, and Bead Milling as Intensification Techniques for Extraction of Lipids from Oleaginous Yarrowia Lipolytica Yeast for a Biojetfuel Application. Bioresour. Technol..

[25] Safi C., Frances C., Violeta A., Laroche C., Pouzet C., Vaca-García C., Pontalier P. (2015). Understanding the Effect of Cell Disruption Methods on the Diffusion of *Chlorella vulgaris* Proteins and Pigments in the Aqueous Phase. Algal Res..

[26] Garoma T., Janda D. (2016). Investigation of the Effects of Microalgal Cell Concentration and Electroporation, Microwave and Ultrasonication on Lipid Extraction Efficiency. Renew. Energy.

[27] Grimi N., Dubois A., Marchal L., Jubeau S., Lebovka N.I., Vorobiev E. (2014). Selective Extraction from Microalgae *Nannochloropsis sp.* Using Different Methods of Cell Disruption. Bioresour. Technol..

[28] Kim D., Vijayan D., Praveenkumar R., Han J., Lee K., Park J., Chang W., Lee J., Oh Y. (2016). Cell-Wall Disruption and Lipid/Astaxanthin Extraction from Microalgae: *Chlorella* and *Haematococcus*. Bioresour. Technol..

[29] Halim R., Harun R., Danquah M.K., Webley P.A. (2012). Microalgal Cell Disruption for Biofuel Development. Appl. Energy.

[30] Singh R.P., Yadav P., Kumar A., Hashem A., Avila-Quezada G.D., Abd_Allah E.F., Gupta R.K. (2023). Salinity-Induced Physiochemical Alterations to Enhance Lipid Content in Oleaginous Microalgae *Scenedesmus sp.* BHU1 via Two-Stage Cultivation for Biodiesel Feedstock. Microorganisms.

[31] Rosine T., Gifuni I., Lavenant L., Pruvost J., Marchal L. (2018). Bead Milling Disruption Kinetics of Microalgae: Process Modeling, Optimization and Application to Biomolecules Recovery from *Chlorella sorokiniana*. Bioresour. Technol..

[32] Carullo D., Demelash B., Alberto A., Donsì F., Perego P., Ferrari G., Pataro G. (2018). Effect of Pulsed Electric Fields and High Pressure Homogenization on the Aqueous Extraction of Intracellular Compounds from the Microalgae *Chlorella vulgaris*. Algal Res..

[33] Shirgaonkar I.Z., Lothe R.R., Pandit A.B. (1998). Comments on the Mechanism of Microbial Cell Disruption in High-Pressure and High-Speed Devices. Biotechnol. Prog..

[34] Zhang X., Yan S., Tyagi R.D., Drogui P., Surampalli R.Y. (2014). Ultrasonication Assisted Lipid Extraction from Oleaginous Microorganisms. Bioresour. Technol..

[35] Cravotto G., Boffa L., Mantegna S., Perego P., Avogadro M., Cintas P. (2008). Improved Extraction of Vegetable Oils under High-Intensity Ultrasound and/or Microwaves. Ultrason. Sonochemistry.

[36] Iqbal J., Theegala C. (2013). Microwave Assisted Lipid Extraction from Microalgae Using Biodiesel as Co-Solvent. Algal Res..

[37] Wahidin S., Idris A., Raehanah S., Shaleh M. (2014). Rapid Biodiesel Production Using Wet Microalgae via Microwave Irradiation. Energy Convers. Manag..

[38] Patil P.D., Gnaneswar V., Mannarswamy A., Cooke P., Munson-mcgee S., Nirmalakhandan N., Lammers P., Deng S. (2011). Optimization of Microwave-Assisted Transesterification of Dry Algal Biomass Using Response Surface Methodology. Bioresour. Technol..

[39] Lam G.P., Postma P.R., Fernandes D.A., Timmermans R.A.H., Vermuë M.H., Barbosa M.J., Eppink M.H.M., Wijffels R.H., Olivieri G. (2017). Pulsed Electric Field for Protein Release of the Microalgae *Chlorella vulgaris* and *Neochloris oleoabundans*. Algal Res..

[40] Goettel M., Eing C., Gusbeth C., Straessner R., Frey W. (2013). Pulsed Electric Fi Eld Assisted Extraction of Intracellular Valuables from Microalgae. Algal Res..

[41] Martínez J.M., Delso C., Angulo J., Álvarez I., Raso J. (2018). Pulsed Electric Fi Eld-Assisted Extraction of Carotenoids from Fresh Biomass of *Rhodotorula glutinis*. Innov. Food Sci. Emerg. Technol..

[42] Zuñiga P.K., Ciobanu F.A., Nuñeza O.M., Stark K.D. (2012). The Use of Direct Transesterification Methods and Autoclaving for Determining Fatty Acid Yields from Dried *Philippine thraustochytrids*, a Potential Source of Docosahexaenoic Acid. J. Funct. Foods.

[43] Obeid S., Beaufils N., Camy S., Takache H., Ismail A., Pontalier P.Y. (2018). Supercritical Carbon Dioxide Extraction and Fractionation of Lipids from Freeze-Dried Microalgae *Nannochloropsis oculata* and *Chlorella vulgaris*. Algal Res..

[44] Sierra L.S., Dixon C.K., Wilken L.R. (2017). Enzymatic Cell Disruption of the Microalgae *Chlamydomonas reinhardtii* for Lipid and Protein Extraction. Algal Res..

[45] Wang M., Chen S., Zhou W., Yuan W., Wang D. (2020). Algal Cell Lysis by Bacteria: A Review and Comparison to Conventional Methods. Algal Res..

[46] Qiu Y., Frear C., Chen S., Ndegwa P., Harrison J., Yao Y., Ma J. (2020). Accumulation of Long-Chain Fatty Acids from *Nannochloropsis salina* Enhanced by Breaking Microalgae Cell Wall under Alkaline Digestion. Renew. Energy.

[47] Nee W., Loke P., Foh C., Tao Y., Chang J. (2018). Improving Cell Disruption Efficiency to Facilitate Protein Release from Microalgae Using Chemical and Mechanical Integrated Method. Biochem. Eng. J..

[48] Satiro J., Gomes A., Florencio L., Simões R., Albuquerque A. (2024). Effect of Microalgae and Bacteria Inoculation on the Startup of Bioreactors for Paper Pulp Wastewater and Biofuel Production. J. Environ. Manage..

[49] Mata T.M., Martins A.A., Caetano N.S. (2010). Microalgae for Biodiesel Production and Other Applications: A Review. Renew. Sustain. Energy Rev..

[50] Thomas W.H., Tornabene T.G., Weissman J. (1984). Screening for Lipid Yielding Microalgae: 1983 Final Report.

[51] Florentino de Souza Silva A.P., Costa M.C., Colzi Lopes A., Fares Abdala Neto E., Carrhá Leitão R., Mota C.R., Bezerra dos Santos A. (2014). Comparison of Pretreatment Methods for Total Lipids Extraction from Mixed Microalgae. Renew. Energy.

[52] Koberg M., Cohen M., Ben-amotz A., Gedanken A. (2011). Bio-Diesel Production Directly from the Microalgae Biomass of *Nannochloropsis* by Microwave and Ultrasound Radiation. Bioresour. Technol..

[53] Mubarak M., Shaija A., Suchithra T. (2015). V Review Article A Review on the Extraction of Lipid from Microalgae for Biodiesel Production. Algal Res..

[54] Muhammad G., Potchamyou Ngatcha A.D., Lv Y., Xiong W., El-Badry Y.A., Asmatulu E., Xu J., Alam M.A. (2022). Enhanced Biodiesel Production from Wet Microalgae Biomass Optimized via Response Surface Methodology and Artificial Neural Network. Renew. Energy.

[55] Drira N., Piras A., Rosa A., Porcedda S., Dhaouadi H. (2016). Microalgae from Domestic Wastewater Facility ’ s High Rate Algal Pond: Lipids Extraction, Characterization and Biodiesel Production. Bioresour. Technol..

[56] Prabakaran P., Ravindran A.D. (2011). A Comparative Study on Effective Cell Disruption Methods for Lipid Extraction from Microalgae. Lett. Appl. Microbiol..

[57] Guldhe A., Singh B., Rawat I., Ramluckan K., Bux F. (2014). Efficacy of Drying and Cell Disruption Techniques on Lipid Recovery from Microalgae for Biodiesel Production. Fuel.

